# Proxima Green: RGB Color Metrics for Turfgrass Phenotyping in Controlled Conditions

**DOI:** 10.3390/s26154816

**Published:** 2026-07-29

**Authors:** Matthew M. Conley, Reagan W. Hejl, Julia Farias, Desalegn D. Serba, Dong Wang, Clinton F. Williams

**Affiliations:** United States Department of Agriculture, Agricultural Research Service (USDA ARS), U.S. Arid Land Agricultural Research Center, Maricopa, AZ 85138, USA

**Keywords:** perceptual color metrics, RGB imaging, CIELAB ΔE, CIELUV, chromatic texture, vegetation color sensing, turfgrass phenotyping, stress detection

## Abstract

Turfgrass phenotyping relies heavily on visual quality (VQ) ratings and RGB indices like DGCI, but these are limited by observer subjectivity, coarse ordinal scales, or ratio formulations that do not reflect perceptual color differences. Hyperspectral and machine-learning tools overcome some limitations but remain costly and difficult to generalize, motivating the need for scalable and interpretable RGB color metrics. We introduce ΔEg, a perceptually anchored CIELAB ΔE distance from an ideal green that provides a continuous and interpretable measure of canopy color evaluated alongside a panel of RGB-derived metrics. A 3 × 3 nitrogen × irrigation greenhouse experiment using hybrid bermudagrass (TifTuf, *Cynodon dactylon* × *C. transvaalensis*) quantified canopy responses with RGB imaging, spectral reflectance, CCM-300 fluorescence, and chlorophyll assays. ΔEg correlated strongly with chlorophyll (r = 0.72), similar to DGCI (r = 0.73), and both exceeded CCM-300 (r = 0.29). HSVi showed the strongest association with VQ (r = 0.84) and was most sensitive to irrigation (*η_p_*^2^ = 0.63). CIELUV v* explained the greatest model variation (R^2^m = 0.94) and responded most to fertilizer (*η_p_*^2^ = 0.84). The yellow fraction was significant across all main and interaction effects and captured canopy decline (r = −0.82 with VQ). An illustrative decision-support scenario using ΔEg indicated that moderate fertilizer combined with mild deficit irrigation optimized turf color and input efficiency. Conclusions apply to controlled conditions, with field-scale validation identified as future work. These results demonstrate that interpretable RGB color metrics, anchored by ΔEg, offer a scalable alternative to VQ scoring and spectral systems.

## 1. Introduction

Traditional digital photography has matured to balance image quality, usability, and affordability, making it an increasingly valuable tool for plant phenotyping [1]. Contemporary systems rely on consumer-grade complementary metal-oxide-semiconductor (CMOS) image sensors, to convert incident light into electrical charge packets through dense arrays of photon-sensitive pixels. Photogenerated charge is subsequently amplified and processed on-chip via integrated analog-to-digital conversion and digital processing. This “camera-on-a-chip” architecture enables high-resolution imaging and accurate color reproduction while maintaining low cost and operational simplicity [2]. These attributes support robust, non-destructive measurement of plant morphological and physiological traits, including leaf area, canopy structure, and colorimetric indicators of stress or nutrient status [3]. Moreover, the perceptual optimization that underpins widespread adoption of digital photography in everyday contexts translates to scientific applications, providing a scalable and cost-effective approach for quantifying vegetation across diverse environments [4,5]. Consequently, the use of RGB cameras has become common in plant phenotyping, enabling high-throughput, non-destructive monitoring of plant traits in both field and controlled settings [6]. They can be mounted on unoccupied aerial systems (UAS) to assess canopy cover and color traits under field conditions [7] and integrated into controlled environments where robotic conveyance transports plants to robust imaging stations [8].

Despite these advantages, RGB systems exhibit inherent limitations. Their reliance on Bayer filter mosaics introduces interpolation artifacts [9], while photographic exposure settings, white balance algorithms, and in-camera tone mapping contribute to imaging variability [10]. Typical conversions to the sRGB color space and lossy JPEG compression further reduce data fidelity [11]. Even when RAW image files and color calibration panels are employed, exact spectral recovery remains unattainable due to the limited dimensionality of the RGB color space and overlapping spectral sensitivities of the camera detectors [12]. Additionally, most consumer-grade cameras incorporate optical filters that block ultraviolet (UV) and near-infrared (NIR) wavelengths, excluding spectrally informative regions relevant to plant physiology [13]. Therefore, these limitations motivate further consideration of how RGB imaging can be most effectively applied in plant phenotyping.

Turfgrass phenotyping has historically relied on subjective visual assessments to evaluate plant health, visual color and quality, and physiological status [14]. Visual quality (VQ) scores capture treatment responses in turfgrass [15] but are limited by observer subjectivity [16], ordinal scaling [17], low-frequency sampling [18] and low reproducibility across environments [19]. Hyperspectral sensing and machine learning approaches overcome most of these limitations but can be costly and computationally intensive and may still be difficult to generalize across experiments or field conditions [20]. Image-based phenotyping provides an opportunity to capture turfgrass responses objectively and with high spatial resolution. RGB cameras with controlled imaging methodology can provide valuable phenotyping data [21,22] and offer a scalable, accessible bridge between subjective visual scoring and advanced spectral methods. Conventional RGB image calculations such as the Dark Green Color Index (DGCI) are useful in turfgrass phenotyping [23], but ratio formulations transform color sensing into values that do not fully represent the human experience of “greenness.”

Therefore, we introduce perceptual green distance (ΔEg) as a continuous interpretable color metric that quantifies deviation from an idealized verdant green within a standardized perceptual color space [24]. ΔEg is based on CIELAB ΔE, where Euclidean distances correspond to the magnitude of perceptual color difference; a value change of 5 units coincides with a visible shift in canopy color [25]. By anchoring ΔEg to a biologically plausible, saturated green characteristic of healthy turfgrass, the metric reflects combined changes in chlorophyll-dominated a*, yellowing-related b*, and lightness (L*) related to canopy density while remaining comparable across imaging sessions. This positions ΔEg as a bridge between subjective VQ scores and conventional RGB-based vegetation indices, offering a transparent, human-interpretable, and biologically grounded phenotyping trait.

The objectives of this study were to (1) develop and evaluate a perceptually grounded RGB-derived metric for turfgrass phenotyping; (2) quantify turfgrass responses to factorial fertilizer and irrigation treatments using a reproducible, controlled-lightbox imaging pipeline and image metrics; (3) benchmark RGB-derived traits against VQ ratings, spectral reflectance-derived vegetative indices, chlorophyll fluorescence, and laboratory chlorophyll assays; and (4) integrate phenotypic responses into an economic decision-support illustration model to show how imaging-based traits could be used to inform turfgrass management.

## 2. Materials and Methods

**Figure 1:**
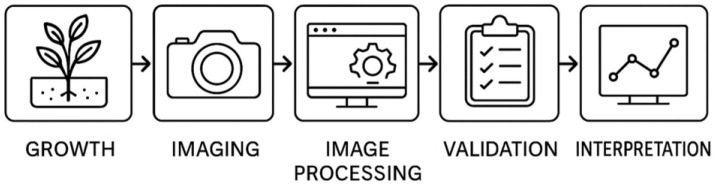
Schematic diagram of the experiment process to examine the interactive effects of irrigation and nitrogen fertilizer on hybrid TifTuf bermudagrass.

An overview of the experimental workflow, including growth, imaging, processing, validation, and interpretation steps is shown in Figure 1.

### 2.1. Experimental Design

A factorial study was conducted in the greenhouse setting to examine interactive effects of irrigation and nitrogen. Hybrid turf bermudagrass (*Cynodon dactylon* × *C. transvaalensis* ‘TifTuf’) was established from stolon cuttings in a greenhouse for 90 days prior to measurements as described in [26]. Plants were grown under treatment conditions for approximately seven weeks, and canopy responses were measured at five time points across a 40-day period (5 October to 14 November 2024). Treatments consisted of three nitrogen fertilizer levels (4.8, 2.4, or 0 g m^−2^; FERT = 1–3) and three irrigation levels (100, 65, or 30% ET_a_; IRR = 1–3), applied in a fully crossed 3 × 3 design. Each treatment combination was replicated across three biological blocks (REP), with canopy responses measured repeatedly on 54 weight lysimeters (LYS); five environmental measurement periods (ENV) were included as a fixed effect factor to capture temporal progression of treatment responses. Two independent greenhouse runs (RUN) helped to quantify background environmental differences and evaluate the robustness of camera-based metrics under slightly differing ambient conditions. Although RUN does not substitute for multi-year replication, it provided an estimate of minor environmental heterogeneity useful for assessing phenotypic metric stability.

Initial experiment findings are described in [26], which reported the primary VQ responses to fertilizer × irrigation treatments. The present study expands analysis by integrating RGB-derived phenomic traits, multisensor validation, and an economic example model, while using the VQ dataset as the primary ground-truth response.

### 2.2. Lightbox Setup and Image Acquisition

A low-cost, fixed-geometry imaging system was used to enable stable and repeatable RGB acquisition. Images were taken inside a lightbox (84 × 52 × 51 cm; L × W × H) that was constructed from cardboard and lined with non-gloss white paper to provide diffuse interior reflectance. Its dimensions were selected to accommodate the lysimeter and plant while keeping box walls outside the camera’s field of view. A removable box top allowed unobstructed access.

A Nikon N1 AW1 camera (Nikon Corporation, Tokyo, Japan) was mounted at the top of the enclosure, positioned 35 cm above the canopy in nadir. Exposure settings were fixed for all samples (ISO 160, f/8, 1/160 s). Custom white balance was set using the lightbox white background under operational lighting. Images were acquired as 4608 × 3072 sRGB JPEGs; RAW files were captured but not used for the primary analysis. Independent verification was performed to confirm that JPEG tone mapping was stable across all frames and did not affect treatment rankings. No post-capture color correction was required because image colors closely matched an X-Rite (X-Rite Incorporated, Grand Rapids, MI, USA) color calibration panel (sRGB RMSE = 2.57; mean ΔE2000 = 4.36) and thus verified stable but non-absolute colorimetry.

Illumination was provided by two strips of flat-spectrum, DC-driven D65 LEDs (CRI 99; Waveform Lighting, Vancouver, WA, USA) delivering ≈ 8000 lux of diffuse, uniform lighting with no emitters directly visible to the camera. Each plant was imaged individually by inserting its PVC (5.2 cm diameter × 30.5 cm height) lysimeter pipe container (with a drained base) through the box floor and into a fixed position. This maintained consistent lighting geometry across sessions.

A hue neutral white background swath within each frame was used to quantify white balance drift and exposure consistency across all images. For every operational session, the neutral patch color differences to pure white (using ΔE2000) remained near zero (session means ≈ 0.0002–0.0767; maximum ≤ 0.4785), corroborating instrumental stability within the lightbox. Minor color deviations are expected in practice because canopy size, geometry, and spectral reflectance all modulate local interreflections; however, reference measurements remained well below perceptual thresholds and did not affect treatment ranking. Although JPEG tone mapping introduces a fixed spectral to RGB transform, the lighting, optics, exposure, and file encoding capture pipeline were held constant. This drove consistent color mapping and enabled robust relative treatment comparisons because HSV threshold sensitivity is inherently stabilized in this controlled pipeline. Sensitivity to outdoor illumination is deferred to planned field validation.

### 2.3. Image Processing and Metric Extraction

#### 2.3.1. Distance from an Ideal Green

**Figure 2:**
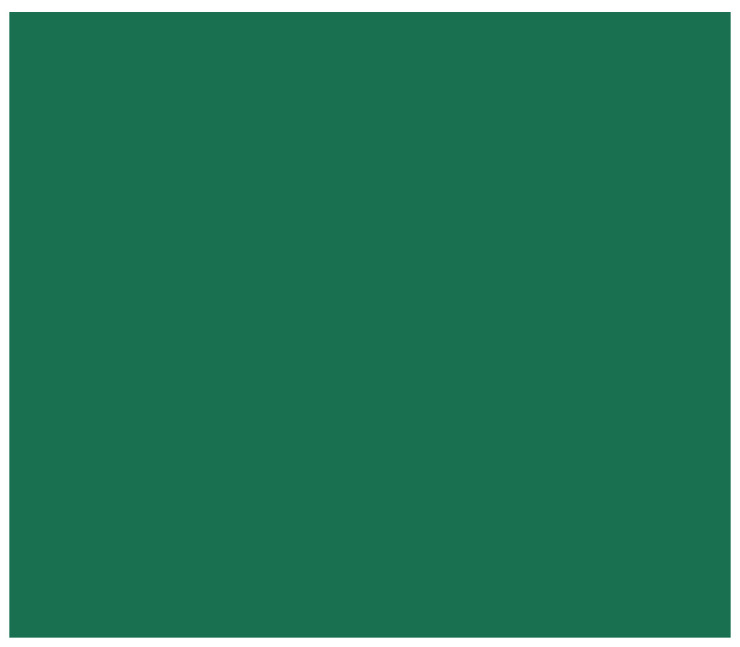
Reference “ideal green” color swatch used as the fixed anchor for ΔEg calculations. RGB (24, 109, 79), HSV (159°, 78%, 43%), CIELAB (40.74, −31.71, 9.90), Hex #186D4F, and Munsell approximation 5G 4/6.

A fixed synthetic “ideal green” was defined to support a perceptually interpretable turfgrass color metric. The reference color (Figure 2) represents a dark saturated viridian-type green associated with visually healthy vegetation and was selected as a stable anchor for color distance-based comparisons. Using a fixed color reference is consistent with color science practice in which synthetic target colors can quantify appearance deviations in a perceptually uniform space [27].

For each canopy pixel, ΔEg was computed as the CIELAB ΔE distance from the reference green. CIELAB (ISO/CIE 11664-4:2019) [28] provides an approximate perceptually uniform metric space, where Euclidean distances correspond to perceived color differences; values near 1 represent possibly noticeable differences, while values greater than 5 are more easily perceptible under typical viewing conditions [11]. ΔEg provides an interpretable, scaled measure of canopy greenness loss relative to the defined ideal. Because CIELAB is a metric space, shifting the reference green would not alter relative treatment comparisons, only the absolute offset. ΔEg thus functions as a unidirectional indicator of color departure analogous to ΔE metrics widely used in appearance quality assessment [27].

The ideal green anchor used for ΔEg is intentionally defined as a synthetic, perceptually meaningful reference color rather than a physiological optimum. Because canopy appearance is influenced not only by pigment concentration but also by leaf angle distribution, texture, and surface scattering, no single color corresponds to a universal physiological state. A fixed color anchor therefore serves as a normalization point, enabling consistent comparison of canopy color perceptual differences across environments and imaging sessions. The specific choice of green does not alter treatment rankings or statistical contrasts because the anchor simply defines the reference scale for perceptual deviation.

#### 2.3.2. Color Filter Mask

Threshold values for the fractional color areas of %Y (yellow), %G (green) and %Gr (greener) were determined iteratively in the HSV color space using an interactive graphical interface and representative sample images, then verified with additional images, similar to the approach used in TurfAnalyzer [29] and following our previous study using consumer-grade photography for turfgrass phenotyping, where an example image segmentation figure can be found [30]. Manual inspection of segmented overlays confirmed correct class allocation across all images. Hue thresholds followed the 1–180 OpenCV circular scale, while saturation and value used the 0–255 linear 8-bit scale. The identical saturation and value thresholds for %G and %Gr reflect that these classes differ only in shade of green. %G pixels were defined by hue values from 30 to 60, with saturation and value ranging from 40 to 255 and 1–255, respectively. The %Gr classification restricted hue to 40–60, isolating deeper green tones with no change in illumination sensitivity. %Y pixels were identified using hue values from 20 to 29, saturation from 80 to 255, and value from 1 to 255.

#### 2.3.3. Color Metric Definitions and Computational Formulations

Five color metrics were calculated exclusively for the pixels classified within the %G range; therefore, they focus representation on the color quality features of the photosynthetically active portion of the canopy, rather than the entire image. Resultant color indexes are independent of the proportion and spatial arrangement of %G, and complementary to the fractional color area calculations. The following equations define the quantitative relationships used in this phenotyping method; they link the image-derived parameters to perceptual color descriptors, pigment-related opponency axes, and fractional canopy composition.

ΔEg (perceptual green difference, Equation (1)) quantifies canopy color deviation from an idealized green (Figure 2) using the CIELAB ΔE perceptual color-difference model. For each pixel classified as %G, ΔEg was computed as

(1) $$\Delta E_{g}=\sqrt{{\left(L^{*}-L_{g}^{*}\right)}^{2}+{\left(a^{*}-a_{g}^{*}\right)}^{2}+{\left(b^{*}-b_{g}^{*}\right)}^{2}}$$

DGCI (Dark Green Color Index, Equation (2)) is a widely used RGB-based indicator of turfgrass greenness derived from normalized transformations of hue, saturation, and brightness value. It correlates with VQ ratings [31,32,33] and chlorophyll concentrations [34,35]. Hue is defined on the traditional 360° scale for this standard formula. DGCI was calculated as

(2) $$DGCI=\frac{\left(\frac{Hue-60}{60}\right)+\left(1-Sat\right)+\left(1-Value\right)}{3}$$

HSVi (Hue difference illumination ratio, Equation (3)) describes shifts in hue that correspond to greening or yellowing. It is based on the DGCI approach and has shown correlation with near-infrared reflection [30]; Hue is defined in the OpenCV 180° scale for this formula. HSVi was calculated as

(3) $$HSVi=\frac{Hue-(Sat/Value)}{40}-0.3$$

BA (Chromatic balance, Equation (4)) is the ratio of the CIELAB b* and a* opponent axes, where a* reflects chlorophyll-related green–red and b* reflects carotenoid-related blue–yellow. BA describes the slope of the chromaticity vector and serves as a luminance-insensitive (*r* = 0.2 in this dataset) indicator of pigment-related color shifts. Previous work has shown that chromatic balance correlates with pigment-sensitive spectral indices such as RedEdge and NDRE [30]. BA was calculated as

(4) $$BA=\frac{b^{*}}{a^{*}}$$

BA_SD_ (Chromatic dispersion, Equation (5)) quantifies the spatial variability of chromatic balance (BA) within the canopy. BA_SD_ captures directional color heterogeneity related to micro-scale variation in pigment composition, leaf orientation, and canopy structure. For each image, BA_SD_ was calculated as the standard deviation of BA across all green-classified pixels:

(5) $$BA_{SD}=\sqrt{\frac{1}{N-1}\sum_{i=1}^{N}{\left(BA_{i}-\bar{BA}\right)}^{2}}$$

%Y (yellow area fraction, Equation (6)) quantifies the proportion of canopy pixels classified as yellow, based on HSV hue thresholds between 20 and 29° (on the 180° scale) and immediately below the %G range [32]. The yellow fraction is commonly associated with stress, senescence, or loss of chlorophyll and typically shows an inverse relationship with turfgrass VQ [9,16,30]. %Y was calculated as

(6) $$\%Y=\frac{N_{yellow}}{N_{total}}\times 100$$

%G (green area fraction, Equation (7)) quantifies the proportion of canopy pixels classified as green based on HSV hue thresholds between 30 and 60° (on the 180° scale), a commonly used range for identifying living green vegetation in turfgrass imaging [31,32,33,34], %G was calculated as

(7) $$\%G=\frac{N_{green}}{N_{total}}\times 100$$

%Gr (greener area fraction, Equation (8)) quantifies the proportion of canopy pixels classified based on HSV hue thresholds between 40 and 60° (on the 180° scale) considered deep or dark green, excluding yellow–green hues to emphasize green tones typically associated with higher turf quality [32,36]. %Gr was calculated as

(8) $$\%Gr=\frac{N_{greener}}{N_{total}}\times 100$$

All metrics were derived using custom Python 3.11.8 scripts [37] NumPy (version 1.26.4), pandas (version 2.0.2), SciPy (version 1.11.4), scikit-image (version 0.25.2), OpenCV (version 4.11.0), Pillow (version 11.3.0), matplotlib (version 3.6.3), and seaborn (version 0.12.2) used for visualization.

### 2.4. Multi-Spectral Measurements

Active optical reflectance measurements were collected using a CS-45 RapidScan handheld sensor (Holland Scientific, Lincoln, NE, USA), which has been employed effectively in turfgrass research [38] and measures Red 670 nm, RedEdge 730 nm, and NIR 780 nm reflectance. The sensor was positioned nadir 60 cm above the grass canopy, and three readings were acquired per lysimeter at each measurement event. Two spectral vegetation indices were computed: the Normalized Difference Vegetation Index (NDVI; Equation (9)), which is sensitive to chlorophyll content and canopy structure, and the Normalized Difference RedEdge index (NDRE; Equation (10)), which enhances sensitivity to pigment variation and nitrogen status through use of the RedEdge band. NDVI and NDRE were calculated as

(9) $$NDVI=\frac{NIR-RED}{NIR+RED}$$

(10) $$NDRE=\frac{NIR-RedEdge}{NIR+RedEdge}$$

### 2.5. Chlorophyll-a and -b Content Measurement

Laboratory extraction and spectrophotometry were used as the biochemical benchmark for chlorophyll content. At weeks 2 and 4 (31 October and 14 November), lysimeters were trimmed to approximately 2.5 cm in height, and half of the plant clippings were collected for pigment analysis. Samples were immediately placed in liquid nitrogen and subsequently stored at −20 °C until analysis. Total chlorophyll content (chlorophyll a + chlorophyll b) in leaves was determined following the method of [39], with calculations based on the adjusted Equations (11) and (12) proposed by [40], where A_665_ and A_649_ represent absorbance at 665 nm and 649 nm, respectively.

Chlorophyll-a = 12.19A_665_ − 3.45A_649_ (11)

Chlorophyll-b = 21.99A_649_ − 5.32A_665_ (12)

Pre-weighed frozen leaves were incubated at 65 °C in dimethyl sulfoxide until complete tissue bleaching was achieved. The sampling frequency was designed to follow natural mowing intervals for turfgrass. Destructive clipping beyond these two growth-driven events would have induced stress and confounded treatment responses. Pearson’s r was applied to the continuous chlorophyll data for linear association.

Optical chlorophyll fluorescence (CHL_s_) was measured non-destructively using a CCM-300 fluorescence sensor (Opti-Sciences, Hudson, NH, USA) calibrated with the factory reference slide. The instrument estimates relative chlorophyll content using the fluorescence ratio method described by [41] as implemented in the CCM-300 firmware [42]. The sensor excites leaf tissue with modulated red and far-red light, then records emitted fluorescence at 700 nm and 735 nm and computes a chlorophyll-sensitive ratio. The optical index was calculated as Equation (13).

(13) $$CHLs=\frac{F_{735}}{F_{700}}$$

### 2.6. Visual Quality Assessment

VQ ratings were collected using the standard National Turfgrass Evaluation Program (NTEP) scoring as described in [26]. VQ is conducted by trained evaluators who assign scores on a 1–9 scale, where 1 indicates dead turf and 9 indicates optimal quality. The rating incorporates visual color, density, uniformity, and overall turf aesthetic. Because VQ is a perceptual evaluation method rather than a numerical computation, no equation is used. Pearson, Spearman, and Kendall correlations were applied to VQ due to its ordinal scale.

### 2.7. Statistical Analysis

Preliminary data tabulation and organization was performed in Microsoft Excel version 2606 (Microsoft Corporation, Redmond, WA, USA). All statistical analyses were conducted in R™ 4.4.1 (R Core Team, Vienna, Austria), using R studio 2024.09.1 Build 394 [43]. Linear mixed-effects primary models were fitted using the lme4 package (version 1.3.6; Bates et al., R Core Team, Vienna, Austria) with significance assessed using Satterthwaite-approximated degrees of freedom as implemented in lmerTest (version 3.2.0; Kuznetsova et al., Copenhagen, Denmark).

Linear mixed-effects models (LMM) included LYS as a random intercept term to account for repeated measurements on each experimental unit. Model specification treated ENV as a categorical fixed effect factor representing sampling dates, capturing the systematic temporal variation across microclimate and lighting conditions without imposing a correlation structure on residuals. Preliminary comparisons of models with autoregressive correlation indicated no improvement in fit once ENV was included. This confirmed that ENV adequately accounted for temporal dependence. RUN was included as a fixed effect to quantify differences between growth in separate greenhouses. Serving as a proxy of growth variation between years for the same season, RUN supported more independent treatment-effect estimations across minor growing condition differences [26]. RUN does not substitute for multi-year replication, but it does provide a measure of variability that strengthens inference by accounting for environmental heterogeneity, and it allows evaluation of the response variable sensitivity to background conditions to highlight which measures were more robust to ambient variation.

All hypothesis tests were two-tailed with α = 0.05. For mixed-model ANOVA tables, Type III sums of squares were used with Satterthwaite-approximated denominator degrees of freedom. Partial Eta Squared (*η_p_*^2^) effect sizes were calculated for each fixed factor in the ANOVA. Daily slopes of perceptual color difference (∆Eg) were calculated using Python 3.11.8 (Python Software Foundation, Wilmington, DE, USA) within Jupyter Notebook 6.4.12 (Project Jupyter, Berkeley, CA, USA). The computing environment was managed using Conda 4.14.0 (Anaconda Inc., Austin, TX, USA). Slope trajectories of ΔEg were visualized using ggplot2 (version 4.0.3; Springer-Verlag, New York, NY, USA) implemented in R.

Residual diagnostics including QQ-plots, residuals vs. fitted, Shapiro–Wilk, Anderson–Darling, KS tests, and autocorrelation of standardized residuals were conducted for all LMMs and indicated approximately normal residuals with no systematic dispersion or temporal autocorrelation. Cluster-level influence was evaluated using Cook’s distance and dfbetas with a 4/*m* cutoff (*m* = number of LYS); refitting models after excluding flagged LYS where present produced identical Type III ANOVA results; no observations were removed.

Pairwise correlation analysis was performed on subsets of the full dataset, comprising 189 samples for VQ observations and 159 samples for CHLs measurements. VQ analysis included Pearson’s product–moment correlation (r), Spearman’s rank correlation (*ρ*), and Kendall’s tau correlation (*τ*). Laboratory-derived chlorophyll concentrations correlation used only Pearson’s method. Confidence intervals for Pearson correlations were calculated using Fisher’s z-transformation. In addition to the correlation analysis, simple linear models were fitted for VQ and laboratory chlorophyll using each RGB-derived index. Model performance was evaluated using 5-fold cross-validation grouped by LYS, and RMSE, MAE, and R^2^ were summarized to assess predictive accuracy. For VQ, an interpretable MAE was calculated by rounding predictions to the 1–9 NTEP scale. All correlation analysis was performed in R.

To evaluate treatment differences within the 5 measurement periods, estimated marginal means (EMMs) were computed for the fertilizer × irrigation combinations using the emmeans package in R (version 2.0.3; Lenth & Piaskowski, Iowa City, IA, USA). Pairwise contrasts among treatments were computed within each ENV, and multiplicity was controlled using Sidak adjustment. Compact letter reports were generated using methods from multcomp (version 1.4.29; CRAN, Vienna, Austria), indicating groups of treatments not significantly different at *α* = 0.05. Statistical graphics were produced in R using the ggplot2 package.

## 3. Results

### 3.1. Mixed Effects Linear Modeling

Mixed effects model ANOVA for response metrics and experimental treatments, including interactions, calculated variances, and effects sizes demonstrated explanatory power across response metrics (Table 1), with highest marginal and conditional R^2^ values observed for the CIELUV v* color axis. Fertilizer (FERT) and sampling environment (ENV) were best explained by CIELUV v*, while irrigation (IRR) was best explained by HSVi. The significant main effects of measurement responses underscored their treatment sensitivity.

Strong and consistent significance of ENV and its interactions indicates that environmental conditions were the primary drivers of variation in treatment responses, consistent with temporal development of the canopy. FERT × ENV was best explained by CIELUV v*, and IRR × ENV was best explained by ∆Eg. The weaker FERT × IRR and FERT × IRR × ENV interactions were best explained by %Gr, and were only significant for %Gr, %Y and BA_SD_. Notably, %Y was the only metric to show significance across all effects and interactions, while BA_SD_ provided an indication of chromatic uniformity showing sensitivity across all interactions. Random effects associated with individual samples accounted for additional variance but did not lessen the strength of the fixed effects.

Residual checks supported model assumptions across all responses; mild tail deviations appeared in some metrics at *n* = 243 but QQ-plots and KS tests indicated acceptable fit, and residual autocorrelation factors were negligible under the ENV-coded measurement dates. Cluster-level influence screening identified few LYS units exceeding the 4/*m* cutoff, but all refitted models yielded identical Type III ANOVA results.

### 3.2. Relationships Between Vegetation Metrics and Visual Quality

Correlations between VQ ratings and vegetation metrics are summarized in Table 2. Positive correlations indicate increasing index values with higher VQ, whereas negative correlations indicate inverse relationships. All indices except CIELUV v* and CHLs showed highly significant associations (*p* < 0.001). HSVi and %Y were the strongest correlates of VQ, with narrow confidence intervals. In contrast, CHLs and CIELUV v* exhibited the weakest relationships, each with wide confidence intervals. Overall, HSVi, %Y, NDVI, and NDRE emerged as the most reliable indicators of VQ.

### 3.3. Associations with Combined Chlorophyll-a and -b

Pairwise Pearson’s r correlations between laboratory-derived chlorophyll-a and -b concentrations and vegetation metrics, along with corresponding 95% confidence intervals, are shown in Table 3. Positive correlations indicate that higher index values are associated with greater chlorophyll content, and negative correlations indicate inverse relationships. All indices except BA_SD_ showed highly significant associations (*p* < 0.001). DGCI and ΔEg were the strongest correlates, followed by HSVi, %Gr, and NDRE, while BA_SD_ and CHLs exhibited weak relationships. Overall, DGCI, ΔEg, and HSVi were the most reliable indications of laboratory measured chlorophyll.

Cross-validated predictive accuracy closely reflected the correlation patterns in Table 2 and Table 3. For VQ, HSVi showed the lowest error (RMSE = 0.621; MAE = 0.463; R^2^ = 0.717), followed by %Y (RMSE = 0.666; MAE = 0.493; R^2^ = 0.697). For laboratory chlorophyll, DGCI achieved the highest accuracy (RMSE = 0.452; MAE = 0.364; R^2^ = 0.586), with ΔEg performing similarly (RMSE = 0.456; MAE = 0.369; R^2^ = 0.567). These results reinforce that ΔEg provides a perceptually interpretable color-distance measure that performs comparably to DGCI for chlorophyll, while HSVi and %Y remain the strongest indicators of VQ.

### 3.4. Treatment-Induced Distance from Green, ΔEg

**Figure 3:**
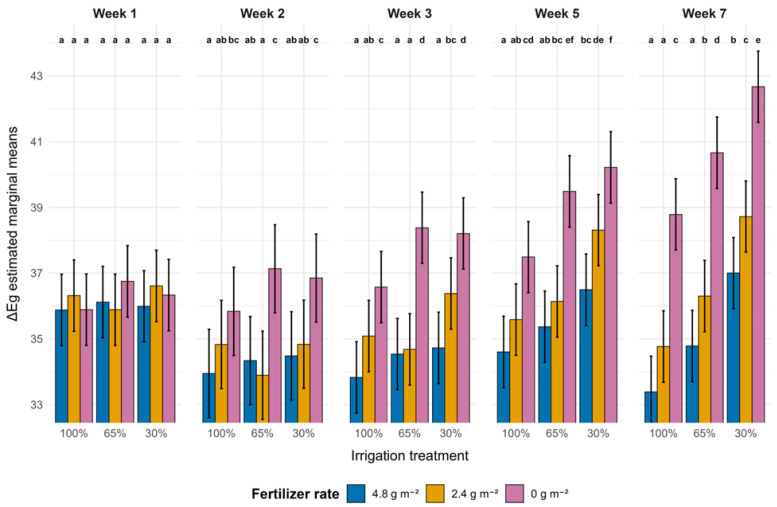
EMMs of ΔEg for all fertilizer × irrigation combinations across the five observation weeks. Higher ΔEg values indicate a greater deviation from the target green color. Bars are grouped by the three irrigation levels (100%, 65%, and 30%), and colors denote the fertilizer rates (4.8, 2.4, and 0 g m^−2^). Panel columns represent the different observation weeks. Error bars show the uncertainty around EMMs (95% CI), and letters above bars indicate statistical groupings from post hoc pairwise comparisons within each week (Sidak-adjusted). Treatments that share the same letter are not significantly different, while treatments with different letters differ at *α* = 0.05.

LMM-derived EMMs of ΔEg reveal clear temporal divergence in turfgrass green color quality among fertilizer × irrigation treatments (Figure 3). Measurement periods are referred to as “weeks” for clarity, although sampling was not performed in weeks 4 and 6 and time progressed continuously between measurement dates.

Initially in week 1, all treatments clustered near ΔEg = 36.2 (SD = 0.3) with no significant differences detected; however, in week 2, significant divergence emerged. FERT1 × IRR1 (high fertilizer, full water) and FERT2 × IRR2 maintained ΔEg values about 34 units distant from the ideal color reference, while FERT3 × IRR2 and FERT3 × IRR3 displayed ΔEg values near 37, and both applied fertilizer treatments improved in color (between 1.5 and 2 ∆Eg units). Treatments with higher irrigation and fertilizer inputs were statistically insignificant from each other, but they were still color-distanced from low-input treatments, and the no-fertilizer-with-water-deficit treatments were significantly distance-increased but only showed a minimal color degradation of 0.5 ∆Eg units.

Week 3 color differences were more pronounced. Only FERT1 × IRR1 treatment improved in color quality, and just by a marginal 0.1 ∆Eg unit. The higher-input treatments FERT1 × IRR1, FERT1 × IRR2, and FERT2 × IRR2 still exhibited ΔEg values near 34, while FERT3 × IRR3 increased to exceed ΔEg 38. Treatments separated into four statistical groups, with FERT3 × IRR2 and FERT3 × IRR3 showing significant separation from the other treatments.

In week 5 the color divergence further widened, and all treatments lost color quality. FERT1× IRR1 retained the highest color quality of ΔEg 34.6, while FERT3 × IRR3 degraded to ΔEg 40.2. FERT3 × IRR2 and FERT3 × IRR3 crossed the 5 ΔEg unit noticeable color difference threshold relative to FERT1 × IRR1 (ΔEg 6.1 and 6.8, respectively) and FERT2 × IRR3 was close (ΔEg 4.9). Treatments spanned six statistical groupings, reflecting a gradient of quality loss associated with reducing inputs.

By the final week 7, color quality improved for FERT1 × IRR1 and FERT1 × IRR2 and FERT2 × IRR1 (between 0.6 and 1.2 ΔEg). A color quality pattern was established where the higher input treatments FERT1 × IRR1, FERT2 × IRR1, and FERT1 × IRR2 maintained the lowest ΔEg values (33.4, 34.8, and 34.8 ΔEg, respectively), FERT2 × IRR2 and FERT1 × IRR3 showed a reduced green quality (36.3 and 37 ΔEg, respectively), FERT2 × IRR3 and FERT3 × IRR1 showed further reductions in green quality (38.7 and 38.8 ΔEg, respectively), while FERT3 × IRR 2 (40.7 ΔEg) was separated from FERT3 × IRR3 with the highest ΔEg of 42.7, representing a total decline of 9.3 ΔEg units. Over the 7 weeks, the noticeable 5 ΔEg threshold for green color quality loss was crossed by FERT1 × IRR3, but not by FERT2 × IRR2.

Results suggest that N fertilizer application was more influential than irrigation in maintaining perceptual turfgrass color and quality of the %G area. Fertilizer consistently prevented noticeable color decline, whereas the absence of fertilizer combined with high irrigation led to the most severe color quality loss.

**Figure 4:**
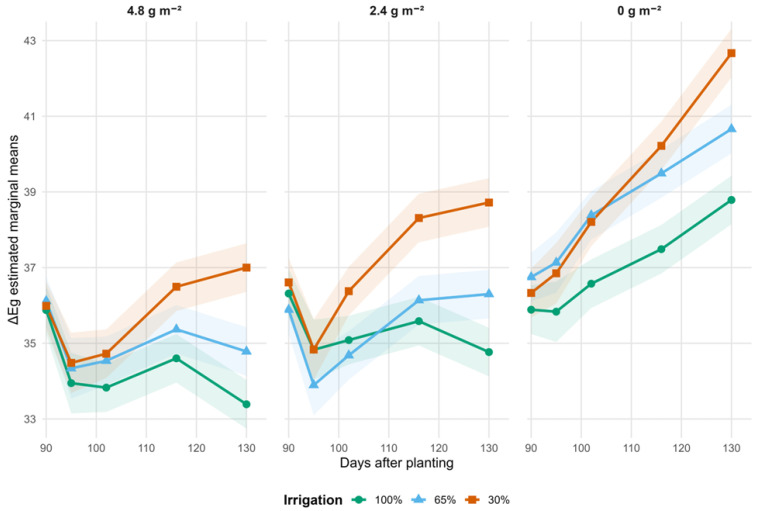
Trajectories of ΔEg EMMs across time for each fertilizer and irrigation treatment combination. Higher ΔEg values indicate greater deviation from ideal green color. Panels show the three fertilizer rates (4.8, 2.4, and 0 g m^−2^), while line colors and point shapes represent irrigation levels (100%, 65%, 30%). Shaded ribbons show the 95% confidence intervals around each EMM trajectory. Points represent the EMM at each observation period, and lines connect these EMMs to illustrate temporal change within each irrigation treatment.

Treatment-specific trajectories of color development are shown in Figure 4. Slopes were plotted against days after planting (DAP) to preserve the true temporal spacing between measurement periods.

The color quality of the %G generally improved for only fully irrigated treatments receiving fertilizer. Although all fertilizer treatments showed an initial improvement across irrigation levels, subsequent weeks revealed color degradation under reduced irrigation, especially in the non-fertilized treatments.

To describe the rate and direction of turf color change, calculated slopes for ΔEg between successive time points using the EMMs for each fertilizer × irrigation treatment were summarized in Table 4 and classified the trend as improving, at plateau, reversing, or declining. At the beginning of the study, almost all treatments exhibited negative ΔEg slopes, indicating early improvement in turfgrass green color. However, these initial gains did not persist for most treatments.

High fertilizer (FERT1) treatments showed the strongest early improvement, with consistently negative initial slopes. As the study progressed, slopes flattened and diverged by irrigation level. By the final interval, the two higher irrigation regimes still exhibited negative slopes, IRR1 (−0.61 ΔEg day^−1^) and IRR2 (−0.29 ΔEg day^−1^), indicating continued late-study improvement while IRR3 transitioned to a positive slope (0.25 ΔEg day^−1^) late in the study, ending with noticeable color loss. Thus, high fertilizer generally sustained color improvement, with decline occurring only under the most severe irrigation deficit.

Moderate fertilizer (FERT2) treatments followed a similar early pattern, with negative slopes during the initial period and then mid-study stabilization. However, late-study responses diverged more strongly than in FERT1. IRR1 remained negative (−0.41 ΔEg day^−1^), indicating continued improvement under full irrigation. In contrast, IRR2 (0.08 ΔEg day^−1^) and IRR3 (0.21 ΔEg day^−1^) shifted to positive slopes, reflecting late-study decline. These results show that moderate fertilizer maintains improvement only when paired with full irrigation.

No fertilizer (FERT3) treatments exhibited the steepest and most consistent positive slopes and showed the strongest decline in green color late in the study. ΔEg increase was most severe under deficit irrigation, with IRR3 (1.23 ΔEg day^−1^); but IRR1 (0.65 ΔEg day^−1^) and IRR2 (0.59 ΔEg day^−1^) also resulted in substantial color deterioration. These patterns demonstrate that in the absence of fertilizer, turfgrass experienced color decline and it was most pronounced under the severe irrigation deficit.

Across fertilizer levels, slope analysis revealed that fertilizer reduced the degree of late-season color decline but did not prevent it. All treatments exhibited positive ΔEg slopes during at least one interval, and most showed positive slopes by the final measurement period. Irrigation influenced the magnitude of color decline, with deficit irrigation amplifying deterioration, particularly under low fertilizer. The most severe late-study losses occurred when low fertilizer and deficit irrigation were combined, highlighting the compounding effects of nutrient and water limitations.

### 3.5. Decision Support Modeling

The ΔEg EMMs and their slope trajectories provided a foundation for a conceptual (not prescriptive) Turf Quality Economic Model (TQEM) decision support framework example that is supplied to show how color metrics can be associated with economic outcomes. The TQEM combines an empirical component, which connects ΔEg units into monetary turf value estimations, and a structural component, which expresses turf quality as a function of fertilizer × irrigation treatments with a risk penalty. However, the TQEM is intentionally simplified and is not intended as a generalizable management tool. Its purpose is to illustrate how an RGB-derived metric such as ΔEg can be translated into economic terms by pairing measured treatment effects with user-defined values for turf quality and input costs. Accordingly, the TQEM results should be interpreted solely as a conceptual demonstration of how RGB-derived color metrics could be incorporated into an economic framework.

The TQEM empirical component mapped final ΔEg onto a linear value function ranging from $5.00 m^−2^ for the best observed color ΔEg 33.3, to $0.00 m^−2^ for ΔEg 50, with intermediate values interpolated proportionally. This mapping represents a user-defined color valuation scheme rather than an attempt to estimate true market prices. Input costs were calculated for the fertilizer application rates and multiplied by a unit price of $0.00132 g^−1^ or $0.6 lb^−1^; and calculated for irrigation levels using the measured average irrigation volume for IRR1 of 6.23 mm day^−1^ × IRR fraction × 40 days (measured period), then multiplied by a municipal commercial irrigation water price of $ 0.0012 L^−1^ or $124 acre inch^−1^. By reporting both empirical return on investment and color quality side by side, the TQEM provides a transparent and adaptable example for balancing input intensity, turf quality, and economic return.

The TQEM structural quality equation is expressed conceptually as (Equation (14)),

(14) $$Value=\alpha \;\mathrm{Water}+\beta \;\mathrm{Fertilizer}-\gamma \;\left(\mathrm{Water}\;\times \mathrm{Fertilizer}\right)-\delta \;\mathrm{Risk}$$

where “Value” is an illustrative scoring metric, “Water” is the irrigation fraction of ET_a_, “Fertilizer” is the application rate (g m^−2^), and “Risk” is the total input cost. The coefficients *α*, *β*, *γ* and *δ* were set a priori (0.8, 0.6, 0.1 and 0.3, respectively) to enhance positive contributions of water and fertilizer, diminishing returns at high input levels, and penalties for monetary cost. For each treatment, the model returned the final ΔEg, its gross turf value per m^2^, the fertilizer and irrigation input costs per m^2^, a total cost per m^2^, and the net value per m^2^. Gross value, cost, and net value results were also scaled by 10,000 to approximate commercial management areas such as a FIFA sports field or short golf par-3 hole with a modest fairway. The TQEM provides a transparent and adaptable example of how to balance input intensity, turf quality, and economic return based on a consumer camera color metric. Although metrics other than ΔEg could have been used to drive the example TQEM, ΔEg was selected for its interpretability and association with VQ.

To avoid complexity and focus solely on the value of turfgrass color, additional factors such as the cost of mowing, equipment maintenance and other realistic management components were not included. Pricing for water and fertilizer, as well as other model parameters, was determined to describe an example situation and should be adjusted for accuracy in any specific scenario. The structure, coefficients, and assumptions of the TQEM are placeholders meant to demonstrate the conceptual pathway from phenotypic change to economic interpretation. Managers or researchers would substitute their own thresholds, nonlinear penalties, step functions, or valuation schemes as appropriate for their specific environments. The TQEM is presented as an example of how a camera measurement can be tied to relational monetary outcomes to support decision-making.

The TQEM illustration shows that under the chosen example coefficients, moderate fertilizer with reduced irrigation produced the highest net values. These strategies balanced acceptable turf quality with lower input costs. High-input treatments achieved the greatest gross turf value, but higher irrigation costs reduced the net returns to levels comparable with moderate-input strategies. In contrast, unfertilized treatments consistently underperformed, and despite lower input costs, their quality losses outweighed savings, resulting in the lowest net values. Fertilizer provision emerged as the dominant driver of turf quality and return on investment, while irrigation adjustments fine-tuned outcomes. Based on this relationship, managers could prioritize fertilizer inputs and adopt moderate irrigation levels to maximize color quality while conserving water, see Table 5.

## 4. Discussion

This study demonstrates that RGB-based metrics can complement or outperform sensor-based proxies in turfgrass phenotyping, offering relatively simple decision-support alternatives to more complex or specialized approaches. By introducing a CIELAB color-distance metric anchored to an ideal reference green (ΔEg), perceptually grounded color-difference modeling is extended into turfgrass imaging, building on prior work showing that ΔE provides an accurate and explicable measure of color-difference magnitude [44]. ΔEg captured treatment effects, was insensitive to the greenhouse used, and showed strong correlation with other key image metrics such as DGCI, VQ, and laboratory chlorophyll. ΔEg is designed to provide a direct quantification of the magnitude of visible color change within a human-sensation color-space framework, translating canopy appearance into ΔE units that reflect how differences in green are perceived. It effectively portrayed how experimental treatments affected turfgrass green color quality and how color changed over time. ΔEg slopes quantified treatment main effects and were used to develop the TQEM, demonstrating how color quality and monetary value can be maintained with reduced water when fertilizer inputs are sufficient.

This study advances RGB-based phenotyping by characterizing several functionally distinct color metrics that extend analysis beyond conventional vegetation indices. Because RGB cameras operate as integrated optical sensors, the demonstrated stability of the controlled-lightbox pipeline and perceptually grounded behavior of metrics such as ΔEg highlight their potential to inform development of low-cost, interpretable plant sensors. Consumer-grade imaging is inherently scalable because it is accessible, inexpensive, and easily expanded to multiple cameras or larger imaging volumes. These results provide practical reference points for designing sensing architectures, calibration procedures, and evaluation benchmarks that translate perceptual color differences into physiologically meaningful signal variations.

While ΔEg served as a continuous, interpretable bridge between subjective visual ratings and established green-color indices (rather than the strongest statistical predictor of VQ or chlorophyll), chromatic dispersion (BA_SD_) expanded color analysis by capturing within-canopy heterogeneity using directional chromatic balance. BA_SD_ quantified color spatial variability in pigment distribution and canopy structure representing patchiness in green to green–yellow composition associated with uneven stress expression. It was sensitive to environment, fertilizer and all treatment interactions, consistent with a texture-like trait influenced by both physiological changes and their spatial distribution. Its moderate correlation with VQ suggested that BA_SD_ detects aspects of canopy uniformity that contribute to perceived quality under moderate stress conditions, when canopies are neither uniformly healthy nor uniformly senescent.

The strong explanatory power of the CIELUV v* blue–yellow opponent axis indicated that phenotypic variation in this study was not fully described by traditional greenness. CIELUV v* showed the highest mixed-effects model explanatory power yet exhibited weak correlation with VQ and moderate correlation with chlorophyll and ∆Eg. This pattern suggests that it responded to biological features sensed by the camera but not emphasized in human-perceived quality, capturing an orthogonal chromatic dimension linked to pigment balance and water-related canopy condition. As nutrient or water limitations increased, CIELUV v* shifted consistently toward the yellow pole, resolving treatment-induced color changes that were distinct from greenness magnitude and providing a complementary perceptual axis to interpret turfgrass responses.

DGCI was strong in explaining treatment fixed effects, showing the highest correlation with chlorophyll content, and a moderate correlation with VQ. This pattern suggests that DGCI is more aligned with turf physiological pigmentation status than VQ ratings. In contrast, HSVi showed increased sensitivity to VQ and fixed effects. It produced the second-highest marginal R^2^ value but was more sensitive to environmental effects than DGCI. HSVi correlated most strongly with VQ and moderately with chlorophyll content, indicating that HSVi could be a valuable complement to DGCI.

Fractional green and greener cover areas were each effective in discerning treatment effects, and their utility was strengthened by the inclusion of fractional yellow areas. The yellow area was the only metric to show significance across all main effects and interactions. It was also strongly correlated with VQ and moderately with chlorophyll. This is consistent with previous findings in which the yellow fractional area exhibited a strong association with visual quality in controlled lightbox imaging [30]. Contrasting %G with %Gr demonstrates how hue-band selection modulates sensitivity. The broader %G band explained more fixed-effect variance and showed stronger ENV and FERT × ENV effects, consistent with the temporal admission of yellow–green transitions. Conversely, narrowing the hue band markedly increased sensitivity to fertilizer and strengthened higher-order dynamics. Importantly, %Gr correlated substantially better with laboratory chlorophyll, indicating that deeper greens track pigment concentration.

The distinction between green-area quality metrics and fractional-area metrics is intentional. Metrics such as ΔEg, DGCI, and HSVi characterize the quality of photosynthetically active tissue, while %G and %Y capture green canopy loss through yellowing. Together these components reflect both the magnitude and nature of stress responses without conflating color quality with canopy area.

The broader applicability of these metrics is supported by their successful use in other agronomic contexts, including recent work where image-derived color metrics were applied to evaluate nutrient-delivery performance in mechanochemically synthesized fertilizers [45]. Collectively, these RGB-based metrics constitute a complementary framework in which canopy color can be characterized through multiple interpretable dimensions that align with both plant physiological processes and human perception.

Beyond treatment effects, fixed-effect patterns further support the stability of the imaging pipeline. Because ENV represented measurement-date events, its large effect sizes reflected the expected temporal progression of treatment responses rather than any instability in the imaging system. In contrast, the small RUN effects indicate consistent camera performance across the two greenhouses.

Sustainable turfgrass management depends on optimal irrigation and fertilization inputs. With nitrogen (N) leaching strongly driven by water application, and losses reaching up to 30% of applied inputs [46], matching fertilization to growth stage and implementing efficient irrigation scheduling can markedly reduce environmental losses while preserving turf quality [47,48]. Temporal analysis of ΔEg canopy color trajectories revealed distinct and biologically meaningful differences in how fertilizer and irrigation influenced turfgrass phenotypic expression over time (Figure 3 and Figure 4).

Fertilizer-driven responses dominated early in the experimental period, corresponding to chlorophyll increases inferred from canopy greenness, as N availability is expected to stimulate pigment synthesis. Conversely, irrigation effects became more pronounced at later stages, where water availability governed the maintenance of canopy integrity and delayed the onset of senescence-related color loss. This temporal separation suggests that nutrient availability primarily determines the establishment of canopy color potential, whereas water availability regulates its persistence under stress conditions [26]. Divergence in ΔEg trajectories supported this interpretation, with early-stage convergence followed by increasing treatment separation as environmental limitations intensified. These results demonstrate that time-resolved phenotyping provides critical insight into treatment dynamics that would be obscured by single time-point measurement, with direct implications for optimizing management strategies under variable resource conditions.

The active optical reflectance indices NDVI and NDRE provided benchmarks for comparison with image metrics. Although the RapidScan CS-45 sensor is easy to use and does not require calibration, it lacks fractional area measures and its index calculations are less interpretable than traditional color imagery. Results show that NDVI and NDRE provided sufficient treatment quantification and moderate correlation with VQ and chlorophyll. This makes the sensor a reasonable choice to supplant imagery in some applications. However, the increased correlations found with VQ and chlorophyll using HSVi and DGCI, the interpretability of ΔEg and fractional color areas, and the chromatic texture potential of BA_SD_ offer advantages that may warrant the additional effort of image collection and processing.

Image-based metrics consistently outperformed the CCM-300 sensor in correlation with laboratory chlorophyll content, suggesting that canopy-level imaging may provide more reliable proxies than the single-leaf contact point surface fluorescence measurements collected by the device. In this study, the CCM-300 showed only weak correlation with laboratory chlorophyll. This weak correlation could be expected because the CCM-300 samples fluorescence from a very small surface area with limited optical penetration of the mesophyll. The result is consistent with reports of reduced performance of fluorescence-based meters on graminoid leaves [42] and may be due to sensor anomaly [49]. In contrast, camera-derived indices such as DGCI and HSVi produced stronger chlorophyll predictions, indicating that consumer-grade image-based methods with basic exposure controls may offer estimates of canopy-level chlorophyll content in turfgrass species.

Phenotypic findings were extended by the example TQEM. Treatments combining moderate fertilizer with reduced irrigation produced high net value, balancing acceptable turf quality with lower input costs. High-input treatments achieved the greatest absolute turf value but incurred higher irrigation costs, which reduced their net returns. In contrast, severely reduced irrigation decreased turf value enough to diminish net returns even when fertilizer was supplied. The optimized moderately reduced irrigation with full fertilizer was only 0.35% more valuable than the full irrigation with full fertilizer treatment, while the moderately reduced fertilizer with full irrigation was 7.56% less valuable than the optimum. Unfertilized treatments consistently underperformed; their quality losses outweighed input savings (40 to 64% less valuable than optimum). The model predicts that the absence of fertilizer could translate into a $10,509 to $16,788 reduction in turf color value for a hypothetical 6350 m^−2^ American football field, when compared to the optimum treatment. Stated differently, the model predicts that supplying full fertilizer and reducing water 35% reduces turfgrass color dollar value by 6% but achieves a cost savings of 53%. The slope trajectories and economic outcomes corroborate that fertilizer provision is essential for maintaining turf color quality, while irrigation can be strategically reduced to conserve water without considerably compromising net color value of the living green area. This integration of biological and economic perspectives provides a framework for site-specific management using color metrics that empowers managers to optimize performance and resource allocation.

The inexpensive flat-spectrum lightbox with basic camera exposure controls used in this study achieved image data stability. Despite omitting post-capture corrections, the approach produced relatively consistent results that agreed with spectral reflectance and VQ measurements and allowed quantification of experiment treatment effects. This supports the simple design technique and suggests a utility for future phenotyping applications.

The approach used in this study aligns with broader calls in plant phenotyping for developing simple and interpretable methods. While specialized hyperspectral instrumentation and powerful machine learning models achieve high internal accuracy, they may lack broad applicability and transparency. The example presented using consumer camera color-based metrics paired with economic modeling suggests that turfgrass research may continue to benefit from new evaluations of accessible color imagery.

## 5. Conclusions

Given the restricted temporal and environmental scope, claims of field-scale practicality are moderated here; our results support feasibility under controlled lighting and treatment regimens and motivate validation across multi-year, multi-site field settings.

This study demonstrated that interpretable image-based metrics from exposure-controlled consumer-grade cameras can serve as tools for turfgrass phenotyping that may outperform traditional sensor-based proxies in predicting physiological traits like chlorophyll content. Several RGB-derived indices accounted for more than 90% of the treatment variance in the mixed-effects models.

The incorporation of these metrics into a decision-support framework revealed that mild deficit irrigation combined with full fertilizer supply can provide an optimal balance of living turf green quality, resource efficiency, and economic return within the conditions tested. These findings underscore the practical potential of color-based imagery as both a research tool and a scalable solution for real-world turfgrass management.

By prioritizing simplicity, interpretability, and physiological relevance, this work advances a framework for turfgrass assessment that spans between experimental precision and actionable field guidance. Future research is needed to validate these methods across diverse genetics and growing conditions, refine chlorophyll estimation protocols, and expand the utility of image-based phenotyping in broader turfgrass research and management applications.

## Figures and Tables

**Table 1:** Type III ANOVA results and partial eta-squared effect sizes (*ηp*^2^) for fertilizer (FERT), irrigation (IRR), sampling event (ENV), greenhouse (RUN), and their interactions across all color and vegetation indices. Degrees of freedom are based on Satterthwaite’s approximation. *ηp*^2^ and Cohen’s f were calculated using noncentral F distributions as implemented in the effectsize package. Marginal (R^2^m) and conditional (R^2^c) values represent variance explained by fixed effects and by the full mixed model, respectively. Significance codes: *** *p* < 0.001, ** *p* < 0.01, * *p* < 0.05, *p* < 0.1.

	%Y	%G	%Gr	DGCI	∆Eg	HSVi	CIELuv v*	BA_SD_	NDVI	NDRE	CHL_s
R^2^m	0.837	0.855	0.803	0.859	0.860	0.883	0.943	0.719	0.845	0.823	0.374
R^2^c	0.883	0.894	0.845	0.926	0.935	0.926	0.978	0.716	0.904	0.894	0.433
FERT	0.512 ***	0.348 ***	0.662 ***	0.832 ***	0.827 ***	0.733 ***	0.836 ***	0.159 *	0.616 ***	0.660 ***	0.343 ***
IRR	0.475 ***	0.393 ***	0.438 ***	0.604 ***	0.573 ***	0.633 ***	0.418 ***	0.015	0.500 ***	0.374 ***	0.141 *
ENV	0.887 ***	0.903 ***	0.839 ***	0.774 ***	0.760 ***	0.915 ***	0.979 ***	0.777 ***	0.887 ***	0.861 ***	0.280 ***
RUN	0.214 ***	0.257 ***	0.101 *	0.038	0.051	0.071	0.060	0.180 **	0.109 *	0.170 **	0.053
FERT × IRR	0.222 *	0.116	0.293 **	0.158	0.182	0.166	0.171	0.254 **	0.181	0.151	0.014
FERT × ENV	0.247 ***	0.405 ***	0.291 ***	0.703 ***	0.762 ***	0.521 ***	0.776 ***	0.263 ***	0.490 ***	0.555 ***	0.135 **
IRR × ENV	0.359 ***	0.532 ***	0.084	0.555 ***	0.612 ***	0.511 ***	0.464 ***	0.184 ***	0.541 ***	0.475 ***	0.060
FERT × IRR × ENV	0.175 *	0.085	0.212 ***	0.102	0.094	0.134	0.107	0.208 **	0.090	0.105	0.071

**Table 2:** Pearson’s r, Spearman’s *ρ*, and Kendall’s *τ* correlations between VQ ratings and vegetation metrics. All tests are two-tailed with *α* = 0.05. Confidence intervals for Pearson’s r are 95% intervals computed using Fisher’s z-transformation.

	Pearson’s r	Spearman’s Rho (*ρ*)	Kendall Tau (*τ*)	r Confidence (Lower)	r Confidence (Upper)
%Y	−0.815	−0.803	−0.663	−0.858	−0.760
%G	0.731	0.727	0.585	0.657	0.791
%Gr	0.722	0.784	0.656	0.646	0.784
DGCI	0.655	0.503	0.393	0.566	0.730
ΔEg	−0.632	−0.467	−0.359	−0.711	−0.538
HSVi	0.840	0.776	0.648	0.792	0.878
CIELUV v*	−0.104	0.020	0.020	−0.243	0.039
BA_SD_	−0.600	−0.702	−0.524	−0.684	−0.501
NDVI	0.799	0.792	0.650	0.741	0.846
NDRE	0.746	0.716	0.581	0.675	0.803
CHLs	0.033	0.020	0.016	−0.110	0.175

**Table 3:** Pearson’s r correlation coefficients between laboratory chlorophyll concentrations and vegetation response metrics. All tests are two-tailed with *α* = 0.05. Confidence intervals for Pearson’s r are 95% intervals based on Fisher’s z-transformation.

	Pearsons r	r Confidence (Lower)	r Confidence (Upper)
%Y	−0.603	−0.694	−0.494
%G	0.451	0.317	0.567
%Gr	0.673	0.578	0.750
DGCI	0.726	0.643	0.792
ΔEg	−0.715	−0.783	−0.629
HSVi	0.687	0.595	0.761
CIELUV v*	−0.575	−0.671	−0.461
BA_SD_	−0.140	−0.290	0.016
NDVI	0.616	0.510	0.705
NDRE	0.661	0.563	0.740
CHLs	0.285	0.135	0.422

**Table 4:** Trends in color change by treatment for each fertilizer × irrigation treatment.

Fertilizer	Irrigation	Overall ΔEg Trend	Slopes Description
FERT1 (high) 4.8 g m^−2^	IRR1 (100% ETa)	Improving (best case)	Early strong negative slope (−0.39 ΔEg day^−1^) flattened to near zero before a marked recovery (−0.61 ΔEg day^−1^). Turf color improved overall to reach highest green quality (33.4 ΔEg).
	IRR2 (65% ETa)	Improving to Plateau	Early strong negative slope (−0.36 ΔEg day^−1^) flattened to near zero before recovering (−0.29 ΔEg day^−1^), whereafter turf color continued to improve slowly.
	IRR3 (30% ETa)	Reversing	Early negative slope (−0.30 ΔEg day^−1^) flattened to near zero before turning positive (0.25 ΔEg day^−1^). Turf color initially improved but then substantially declined.
FERT2 (moderate)	IRR1	Improving	Early negative slope (−0.30 ΔEg day^−1^) flattened to near zero before a strong recovery by Week 7 (−0.40 ΔEg day^−1^). Turf color improved and ended near FERT1 × IRR2.
	IRR2	Plateau	Early strong negative slope (−0.40 ΔEg day^−1^) stabilized to slightly positive (0.07 ΔEg day^−1^). Slow color quality degradation.
	IRR3	Reversing	Early strong negative slope (−0.35 ΔEg day^−1^) reversed to positive (0.21 ΔEg day^−1^). Turf color quality declined and ended similar to FERT3 × IRR1.
FERT3 (none)	IRR1	Declining	Early flat slope turned slightly positive (0.05 ΔEg day^−1^) before substantially positive (0.59 ΔEg day^−1^). Turf color quality decreased despite full irrigation.
	IRR2	Declining	Slightly positive slope (0.06 ΔEg day^−1^) turned substantial (0.59 ΔEg day^−1^). Turf color quality decrease accelerated.
	IRR3	Declining (worst case)	Consistently positive slopes (0.10 ΔEg day^−1^) turned most positive (1.22 ΔEg day^−1^) and ended with the worst color quality (42.7 ΔEg). Severe divergence from ideal green over time.

**Table 5:** Turf Quality Economic Model outcomes for each fertilizer × irrigation (FERT × IRR) treatment. Treatments consisted of three nitrogen fertilizer levels (4.8, 2.4, or 0 g m^−2^; FERT = 1–3) and three irrigation levels (100, 65, or 30% ET_a_; IRR = 1–3). Values represent mean ΔEg color quality, associated fertilizer and irrigation input costs, total cost per m^2^, and resulting net economic value. Scaled estimates for a 10,000 m^2^ turf area are provided to illustrate treatment-level differences in a hypothetical operation economic return. Higher net values indicate more favorable combinations of turf color quality and input efficiency.

**Treatment**	**ΔEg**	**Turf Value (m^2^)**	**Fertilizer Cost (m^2^)**	**Irrigation Cost (m^2^)**	**Total Cost (m^2^)**	**Net Value (m^2^)**	**Turf Value (10,000 m^2^)**	**Cost** **(10,000 m^2^)**	**Net Value** **(10,000 m^2^)**
FERT1 × IRR2	34.3	$4.70	$0.006	$0.58	$0.59	$4.11	$47,006	$5895	$41,111
FERT1 × IRR1	33.3	$5.00	$0.006	$0.90	$0.90	$4.10	$50,000	$9035	$40,965
FERT2 × IRR1	34.3	$4.70	$0.003	$0.90	$0.90	$3.80	$47,006	$9003	$38,003
FERT1 × IRR3	36.8	$3.95	$0.006	$0.27	$0.28	$3.68	$39,521	$2755	$36,766
FERT2 × IRR2	36.0	$4.19	$0.003	$0.58	$0.59	$3.61	$41,916	$5863	$36,053
FERT2 × IRR3	38.7	$3.38	$0.003	$0.27	$0.27	$3.11	$33,832	$2723	$31,109
FERT3 × IRR1	38.8	$3.35	$0.000	$0.90	$0.90	$2.46	$33,533	$8971	$24,562
FERT3 × IRR2	40.9	$2.73	$0.000	$0.58	$0.58	$2.14	$27,246	$5831	$21,414
FERT3 × IRR3	44.2	$1.74	$0.000	$0.27	$0.27	$1.47	$17,365	$2691	$14,674

## Data Availability

Data and Python scripts used for image processing and %G, %Gr, %Y, ΔEg, DGCI, HSVi, BA_SD_, CIELUV v* metric computation, and R scripts used for statistical analysis are be available in the USDA National Agricultural Library Ag Data Commons (https://agdatacommons.nal.usda.gov/), Data for—Proxima Green: RGB Color Metrics for Turfgrass Phenotyping in Controlled Conditions, accessed on 27 July 2026. The full RGB imagery archive will be made available upon reasonable request.

## Abbreviations

The following abbreviations are used in this manuscript:

%G	Green Area Fraction
%Gr	Greener (Deep Green) Area Fraction
%Y	Yellow Area Fraction
a*	CIELAB Position Between Green and Red
ANOVA	Analysis of Variance
b*	CIELAB Position Between Blue and Yellow
BA	Chromatic Balance
BASD	Chromatic Dispersion
CHLs	Chlorophyll Fluorescence Ratio Index (F735/F700)
CIELAB	Commission Internationale de l’Éclairage Lab* Color Space
CIELAB ΔE	Color Difference Value Calculated Using the CIELAB (1976) Formula
CIELUV	Commission Internationale de l’Éclairage Luv* Color Space
CMOS	Complementary Metal-Oxide-Semiconductor
D65 LEDs	Standard Daylight 6500 K Light-Emitting Diodes
DAP	Days After Planting
DGCI	Dark Green Color Index
EMMs	Estimated Marginal Means
ENV	Environmental Measurement Period
ETa	Actual Evapotranspiration
FERT	Fertilizer Treatment Levels (4.8, 2.4, or 0 g m^−2^; FERT = 1–3)
HSVi	Hue Difference Illumination Ratio
IRR	Irrigation Treatment Levels (100, 65, or 30% ETa; IRR = 1–3)
L*	CIELAB Lightness
LMM	Linear Mixed Model
LYS	Lysimeter
NDRE	Normalized Difference Red Edge Index
NDVI	Normalized Difference Vegetation Index
NIR	Near-Infrared
PVC	Polyvinyl Chloride
R^2^c	Conditional R-squared
R^2^m	Marginal R-squared
REP	Replication Block
RGB	Red–Green–Blue Color Space
RMSE	Root Mean Square Error
RUN	Greenhouse Run
SD	Standard Deviation
TQEM	Turf Quality Economic Model
UAS	Unoccupied Aerial Systems
v*	CIELUV Position Between Blue and Yellow
VQ	Visual Quality
ΔE2000	Color Difference Value Calculated Using the CIEDE2000 Formula
ΔEg	Perceptual Green Difference (CIELAB ΔE from ideal green)
